# The Relevance of the UPS in Fatty Liver Graft Preservation: A New Approach for IGL-1 and HTK Solutions

**DOI:** 10.3390/ijms18112287

**Published:** 2017-10-31

**Authors:** Arnau Panisello-Roselló, Eva Verde, Mohamed Amine Zaouali, Marta Flores, Norma Alva, Alexandre Lopez, Emma Folch-Puy, Teresa Carbonell, Georgina Hotter, René Adam, Joan Roselló-Catafau

**Affiliations:** 1 Experimental Hepatic Ischemia-Reperfusion Unit, Institut d’Investigacions Biomèdiques de Barcelona (IIBB), Spanish National Research Council (CSIC), 08036 Barcelona, Catalonia, Spain; arnau.panisello.rosello@gmail.com (A.P.-R.); daminzaouali12@yahoo.fr (M.A.Z.); emma.folch@iibb.csic.es (E.F.-P.); ghcbam@iibb.csic.es (G.H.); 2 Faculty of Biology, Universitat de Barcelona, 08028 Barcelona, Catalonia, Spain; e.vaaverde@gmail.com (E.V.); mfloreso10@alumnes.ub.edu (M.F.); nvalva@ub.edu (N.A.); tcarbonell@ub.edu (T.C.); 3 Centre Hépato-Biliaire, AP-PH, Hôpital Paul Brousse, 94800 Paris, France; alexandregl.lopez@gmail.com (A.L.); rene.adam@aphp.fr (R.A.)

**Keywords:** ubiquitin proteasome system, cold ischemic injury, fatty liver preservation, IGL-1, HTK, ATP, AMPK and nitric oxide

## Abstract

The 26S proteasome is the central proteolytic machinery of the ubiquitin proteasome system (UPS), which is involved in the degradation of ubiquitinated protein substrates. Recently, UPS inhibition has been shown to be a key factor in fatty liver graft preservation during organ cold storage using University of Wisconsin solution (UW) and Institute Georges Lopez (IGL-1) solutions. However, the merits of IGL-1 and histidine-tryptophan-ketoglutarate (HTK) solutions for fatty liver preservation have not been compared. Fatty liver grafts from obese Zücker rats were preserved for 24 h at 4 °C. Aspartate aminotransferase and alanine aminotransferase (AST/ALT), glutamate dehydrogenase (GLDH), ATP, adenosine monophosphate protein kinase (AMPK), e-NOS, proteasome activity and liver polyubiquitinated proteins were determined. IGL-1 solution prevented ATP breakdown during cold-storage preservation of steatotic livers to a greater extent than HTK solution. There were concomitant increases in AMPK activation, e-NOS (endothelial NOS (NO synthase)) expression and UPS inhibition. UPS activity is closely related to the composition of the solution used to preserve the organ. IGL-1 solution provided significantly better protection against ischemia-reperfusion for cold-stored fatty liver grafts than HTK solution. The effect is exerted through the activation of the protective AMPK signaling pathway, an increase in e-NOS expression and a dysregulation of the UPS.

## 1. Introduction

Misfolded or damaged proteins are degraded by cells without the non-specific destruction of essential cell constituents [1]. In eukaryotic cells, the proteasome controls protein degradation. Proteins that will be degraded are firstly labelled by ubiquitin in an energy-requiring process named the ubiquitin proteasome system (UPS) [2]. The UPS is identified as a highly specialized degradation machinery compartmentalized by different structural and functional proteins before efficient removal [1]. The 26S proteasome is composed of a core 20S particle, in which proteins are digested to short peptides, and one or two 19S regulatory particles that are responsible for substrate recognition and transport into the core particle [3]. These particles are known to participate in degradation and folding of many regulatory proteins that are involved in cell cycle, metabolism, inflammation and growth, and in other processes including ischemia-reperfusion injury (IRI) [4,5].

IRI is inherent to liver transplantation [6,7]. After organ recovery from the donor, the liver graft is stored in an organ preservation solution in hypothermic conditions in order to maintain graft viability until transplantation. The deprivation of oxygen and nutrients to the organ during this cold storage leads to a severe ATP energy-metabolism breakdown, which is accompanied by concomitant activation of protective cell-signaling mechanisms such as adenosine monophosphate protein kinase (AMPK) [8,9,10]. AMPK acts as a metabolic fuel gauge to restore cellular and whole-body energy balance. Enhancement of AMPK activity also induces e-NOS (endothelial NOS (NO synthase)) activity and thus NO production, which has been widely demonstrated to prevent endothelial cell damage [5].

Cold ischemic storage involves an inherent injury in the preserved grafts, which is partially mitigated by the use of organ preservation solution. The most-used preservation solution in liver transplantation is University of Wisconsin solution (UW), considered the gold standard for transplantation of abdominal organs [11]. Other solutions such as Celsior, histidine-tryptophan-ketoglutarate (HTK) and Institute Georges Lopez (IGL-1) are also widely used. Briefly, UW contains hydroxyethyl starch (HES) as oncotic agent, lactobionate and raffinose (for edema prevention), the antioxidants allopurinol and reduced glutathione, and high potassium and low sodium concentrations [12]. IGL-1 is identical to UW, except that HES is replaced by polyethylene glycol 35 (PEG 35) and has inverted sodium/potassium ratio, as shown in Table 1 [13]. For their part, HTK and Celsior have no oncotic agent.

Recent investigations have shown that the UPS is involved in cold liver IRI [14]. Majetschak’s group reported that ATP activates the 26S proteasome during cold ischemia and increases myocardial injury, and that proteasome inhibition may be a promising tool for improving heart preservation strategies [15]. Following on from that study, our group further demonstrated that the use of UPS inhibitors such as MG132 and bortezomib protected liver graft against cold IRI when used at low doses as additives in preservation solutions and in experimental liver transplantation [16,17]. However, the research into the role of the UPS in liver-graft preservation is scarce and is limited to the use of UW and IGL solutions; much less is known about the use of other preservation solutions such as HTK. With this in mind, the present study focuses on the relevance of the UPS dysregulation associated with liver ischemic injury which occurs when fatty liver grafts are preserved in HTK or IGL-1 solutions, and aims to identify and discuss a variety of protective preservation mechanisms.

## 2. Results

**Figure 1:**
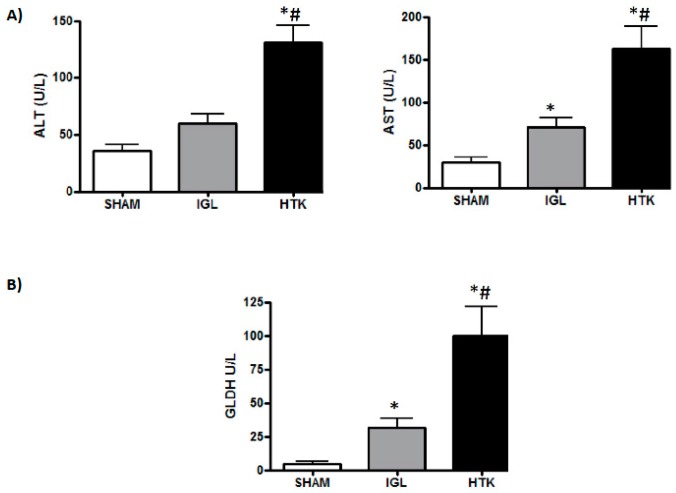
Liver injury (**A**) and mitochondrial damage (**B**) in fatty liver grafts preserved in Institute Georges Lopez (IGL-1) and histidine-tryptophan-ketoglutarate (HTK) vs. SHAM. Results are expressed as mean ± SEM (*n* = 6). * *p* < 0.05 represent significant differences vs. SHAM and # *p* < 0.05 vs. IGL-1.

**Figure 2:**
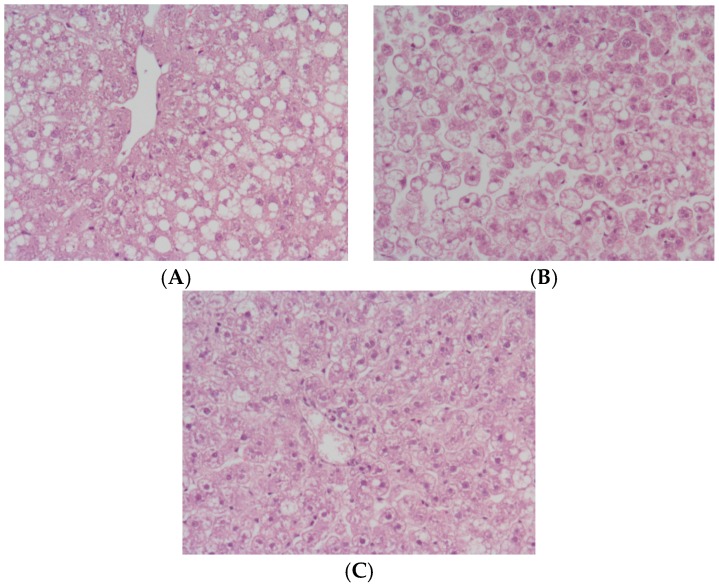
Histology hematoxylin–eosin staining. Photomicrographs of livers at 20× magnification: (**A**) Control group showing normal hepatic architecture with macro- and micro-vesicular fatty infiltration; (**B**) HTK group. Extensive areas of cell dissociation, cell swelling and disintegration of hepatic architecture are seen (**C**) IGL group. Well-preserved lobular architecture with minimal sinusoidal dilatation and cell swelling are observed.

First we evaluated hepatic injury according to transaminase and glutamate dehydrogenase (GLDH) levels, as shown in Figure 1. The release of transaminases and GLDH after 24 h of cold ischemia was more-effectively prevented in IGL-1 solution-preserved fatty livers than in HTK-preserved livers. This was consistent with the histological findings shown in Figure 2. Controls showed grade II to III of fatty infiltration, with hepatocyte integrity maintenance that contrasted with IGL-1 and HTK groups that revealed the most-severe histological changes. HTK group showed severe cellular damage and disintegration of hepatic cords when compared to IGL-1 (Figure 2).

These results were consistent with the higher prevention of mitochondrial damage, determined by GLDH activity levels, in livers preserved in IGL-1 solution than in livers preserved in HTK. The histologic study of control steatotic livers showed grade II to III of fatty infiltration, with hepatocyte integrity maintenance. The other groups conserved fatty infiltration. The most severe changes in histology were observed in the HTK group, showing severe cellular damage and disintegration of hepatic cords. Improved histological characteristics were noted in the IGL-1 group, where moderate cellular damage was seen (Figure 2). Our results indicate that IGL-1 solution was more efficient than HTK solution in reducing hepatic injury and functionality.

**Figure 3:**
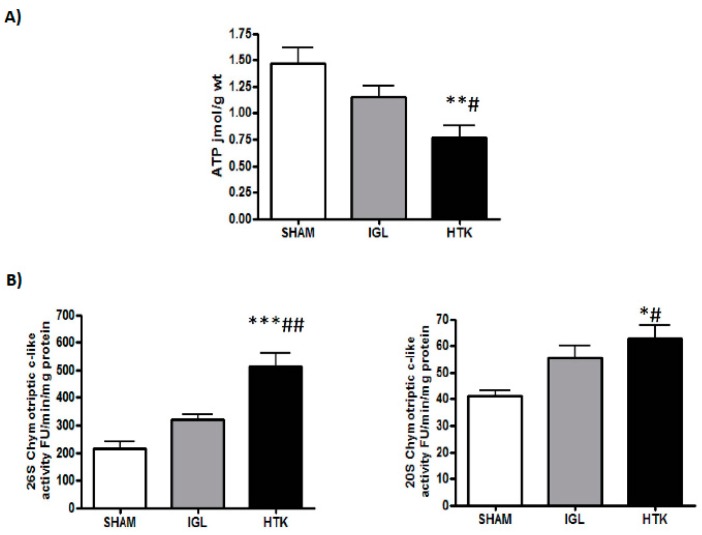
Energy breakdown (ATP failure) (**A**) and chymotriptic-like activity ATP-dependent (26S) and ATP-independent (20S) (**B**) determination. Results are expressed as mean ± SEM (*n* = 6). * *p* < 0.05, ** *p* < 0.01 and *** *p* < 0.001 represent significant differences versus SHAM and # *p* < 0.05, ## *p* < 0.01 versus IGL-1.

**Figure 4:**
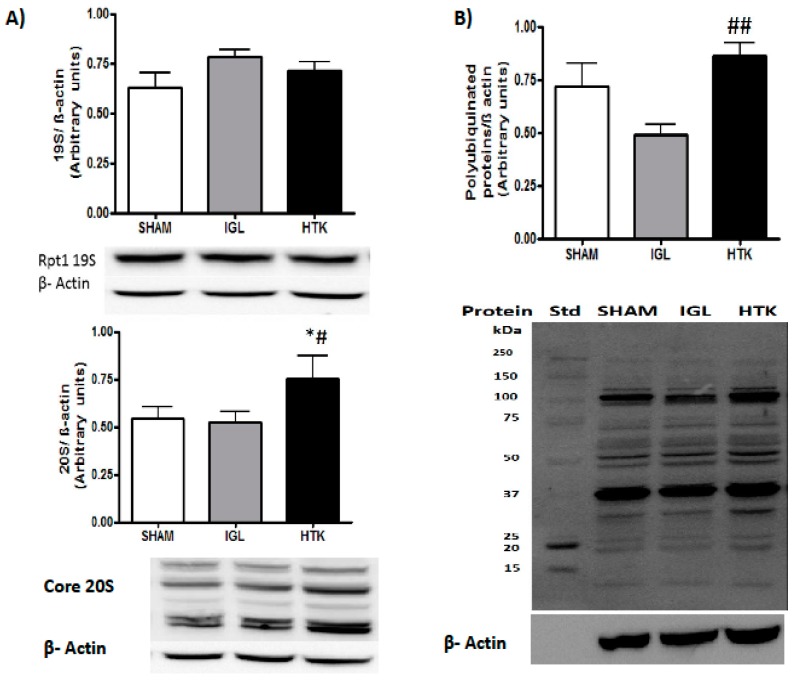
Proteasome 20S and 19S (**A**) and ubiquitinated protein level changes (**B**) in liver grafts preserved in IGL-1 and HTK solutions vs. SHAM. Results are expressed as mean ± SEM of *n* from 3 to 6 (SHAM *n* = 3; IGL-1 *n* = 6 and HTK *n* = 5). (**A**) Representative Western blots of 20S and 19S at the bottom and densitometric analysis at the top. * *p* < 0.05 versus SHAM and # *p* < 0.05 versus IGL-1. (**B**) The blot for ubiquitinated protein detection was stripped (using a mild stripping protocol) and reprobed for β-actin (using a different secondary, anti-rabbit for ubiquitin, anti-mouse for β-actin). Representative Western blots of ubiquitin at the bottom and densitometric analysis at the top. ## *p* < 0.01 versus IGL-1.

We next determined the energy-metabolism breakdown and UPS activity in liver grafts preserved in the two solutions. Livers preserved in IGL-1 revealed higher ATP levels than those preserved in HTK (Figure 3A). This better conservation of ATP levels was associated with a stronger inhibition of UPS activity (measured as chymotryptic-like activity) in fatty liver grafts preserved in IGL-1 solution than in those preserved in HTK after 24 h of cold preservation (Figure 3B). These results are consistent with the higher levels of 20S and polyubiquitinated proteins found in liver grafts preserved in HTK than in grafts preserved in IGL-1 solution (Figure 4). No differences were found in 19S.

**Figure 5:**
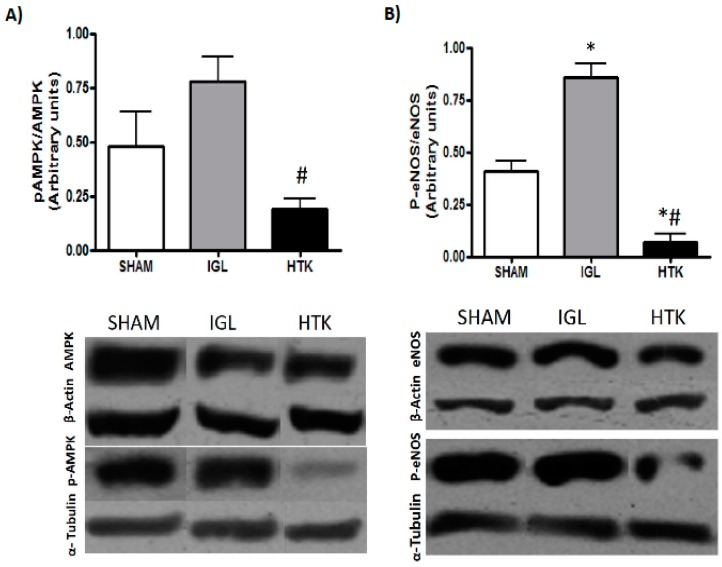
p-AMPK/AMPK (**A**) and p-e-NOS/e-NOS (**B**) protein levels in steatotic liver grafts preserved in IGL-1 and HTK solutions vs. SHAM. Results are expressed as mean ± SEM (*n* = 6). (**A**) # *p* < 0.01 represent significant differences versus IGL-1. (**B**) # *p* < 0.01 represent significant differences versus IGL-1. * *p* < 0.01 represent significant differences versus SHAM.

Finally, in cold-stored livers, we determined the activation of AMPK and e-NOS, two cyto-protective factors induced during ischemic conditions [10,18]. Both tissue cyto-protective markers were expressed more in steatotic livers preserved in IGL-1 solution than in livers preserved in HTK solution, showing a similar profiling pattern (Figure 5). Therefore, IGL-1 provides better protection of fatty livers against the deleterious effects of cold IRI than does HTK.

## 3. Discussion

This paper studied the relevance of the UPS in cold-preserved fatty liver graft injury, comparing two frequently used preservation solutions: IGL-1 and HTK. Our data demonstrated that IGL-1 preservation solution reduced fatty liver injury associated with cold ischemia to a greater extent than HTK solution.

During cold storage, liver grafts are deprived of oxygen and nutrient supply. This stops energetic metabolism, depletes ATP levels and renders the organ more susceptible to IRI [11]. The process is exacerbated in steatotic livers as a consequence of abnormal fat accumulation in hepatic sinusoids, leading to a severe obstruction of hepatic flow with substantial alterations in liver microcirculation [19,20]. In our study, the cold storage of fatty liver grafts in IGL-1 solution significantly prevented hepatocellular and mitochondrial injury in comparison with HTK solution. ATP levels were also better conserved in the fatty livers undergoing IGL-1 solution cold storage.

The AMPK signaling pathway is activated during stress conditions such as exercise, starvation, hypoxia and ischemia, when energy levels are swiftly depleted. Upon energy depletion, AMPK triggers ATP-producing pathways while inhibiting ATP-consuming pathways, hence balancing the metabolic processes towards cellular energy homeostasis [8]. We found a significant activation of AMPK in fatty livers preserved in IGL-1 solution but not in those preserved in HTK solution. This result was consistent with the increased ATP levels found in the fatty livers preserved in IGL-1.

**Figure 6:**
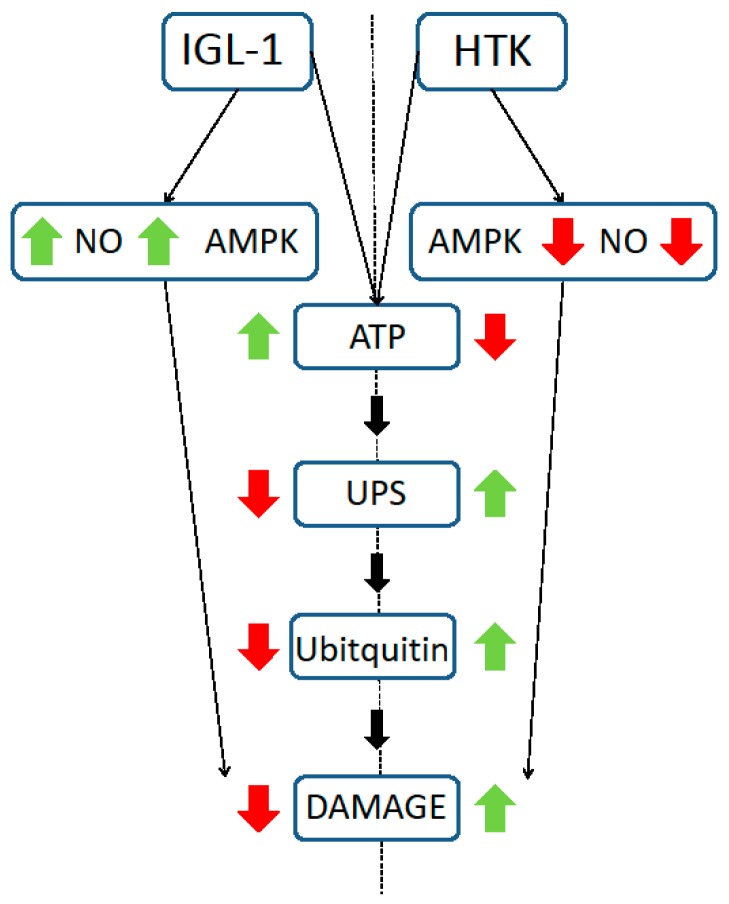
Differential protective mechanisms in fatty liver preservation during graft cold storage. Increases (green arrows) and decreases (red arrows) are represented at each side of the scheme (left side corresponding to IGL-1 solution and right side corresponding to HTK solution).

The direct regulatory effects of ATP on the 26S proteasome have already been described, since ATP is required for 26S proteasome formation and stabilization [21]. It has been shown that both in cell extracts and in proteasomes purified from different sources, ATP depletion can release the 19S regulatory particle from the 20S core, while the addition of ATP can reverse the process [22,23]. These results suggest that under conditions in which the levels of ATP may be compromised, the proteasome activity is likely to be due to the 20S activity [24,25]. More interestingly, in addition to the ATP requirement for the 19S and 20S assembly, proteasome activity increases when the ATP concentration is below physiological levels [15]. This implies that the pathological conditions associated with a decrease in cellular ATP, such as liver cold ischemia, may increase proteasome activity. Accordingly, in our study, the chymotryptic-like activity of 26S and 20S showed that both were significantly increased in fatty livers cold-preserved with HTK solution, precisely the ones with the lowest ATP levels. Furthermore, in this group, the levels of polyubiquitinated proteins were also significantly increased compared with the IGL-1 group. Our findings suggest that IGL-1 provides better protection for fatty livers against the deleterious effects of cold IRI than does HTK (Figure 6).

A key observation in a previous study by our group was the fact that the benefits of IGL-1 solution are associated with the generation of nitric oxide (NO), a potent vasodilator agent which counterbalances the exacerbated microcirculation alterations present in steatotic liver grafts [18,19,26,27]. We have also reported that AMPK activation induces NO synthesis, thus protecting against hepatic injury in both steatotic and non-steatotic liver transplantation [28]. Accordingly, we found a significantly higher expression of e-NOS in fatty livers preserved in IGL-1 solution than in those preserved in HTK.

## 4. Material and Methods

### 4.1. Animals

Homozygous (obese (Ob)) Zücker rats aged 16–18 weeks from Charles River (France) were used. All animals had free access to water and dry food. All animals were used in accordance with protocols approved on 14 July 2016 (483116). The study was performed in accordance with the European Union Directive 2010/63/EU for animal experiments and approved by the Ethics Committees for Animal Experimentation of the University of Barcelona (Directive 396/12). Animals were randomly distributed into groups as described below.

### 4.2. Experimental Groups

Experimental groups were as follows:

- Group 1 (SHAM; *n* = 6): Animals underwent transverse laparotomy and received silk ligatures in the right suprarenal vein, diaphragmatic vein, and hepatic artery.
- Group 2 (IGL-1; *n* = 6): After organ recovery, the livers were flushed with 40 mL of IGL-1 solution and stored in IGL-1 preservation solution for 24 h at 4 °C.
- Group 3 (HTK; *n* = 6): After organ recovery, the livers were flushed with 100 mL of HTK solution (2.5 times more than IGL-1) and stored in HTK solution for 24 h at 4 °C.

After cold storage, the liver specimens in IGL-1 and HTK solutions were washed with Ringer lactate solution (20 mL) and samples were taken from the flush. Liver samples were then stored at −80 °C for the subsequent biochemical determinations.

### 4.3. Biochemical Determinations

#### 4.3.1. Proteasome Chymotryptic-Like Activity Assay

In order to evaluate the proteasome activity, we determined the proteasome chymotryptic-like (β5) ATP-dependent (26S) and non-ATP-dependent (20S) activity using the substrate *N*-Suc-Leu-Leu-Val-Tyr-aminomethylcoumarin from ENZO Life Science (Alcobendas, Madrid), as previously reported [16]. Liver cell lysates were prepared by homogenization in 50 mM Tris, 1 mM EDTA, 5 mM MgCl_2_, 150 mM NaCl, and 1 mM DTT, pH 7.5. The samples were then centrifuged at 12,000× *g* for 15 min (4 °C) and the supernatants were collected. To analyze the proteasome activity of liver homogenates (25 μg/sample), the fluorescent substrate Suc-LLVY-AMC 100 μM for β5 was used. All the assays were carried out in a total volume of 100 μL. The ATP-dependent 26S was measured in the presence of 0.1 mM ATP. Each assay was conducted in the absence and presence of the specific proteasome inhibitor epoxomicin (Peptides International) at a final concentration of 20 µM.

The cleavage products 7-amino-4-methycoumarin (AMC) were measured by fluorimetric techniques (excitation/emission 380/460 nm) according to the supplier’s instructions using a microplate fluorometer (Tecan Infinite Microplate Reader).

#### 4.3.2. Transaminase Assay

Hepatic injury was evaluated by alanine aminotransferase (ALT) and aspartate aminotransferase (AST) levels using commercial kits from RAL (Barcelona, Spain), as previously reported [18]. Briefly, 100 μL of effluent washout liquid or perfusate was added to 1 mL of the substrate provided by the commercial kit, and then transaminase activity was measured at 340 nm with a UV spectrometer and calculated following the supplier’s instructions. Results were normalized using a commercial calibrator (Biocal, RAL).

### 4.4. Histology

Liver samples were fixed in 10% neutral buffered formalin and embedded in Paraplast, and 5-µm sections were stained with hema-toxylin and eosin according to standard procedures. Histologic evaluation was graded semiquantitatively from 0 (no damage) to 4 (severe cellular damage, such as vacuolization, cell dissociation, cell swelling and disintegration of hepatic architecture).

### 4.5. Glutamate Dehydrogenase Activity

Mitochondrial damage was measured as glutamate dehydrogenase activity (GLDH), as previously reported [18], with GLDH Randox Laboratories Ltd. commercial kit (Antrim, UK), using eluate as sample and quantifying the decrease in absorbance at 340 nm according to the manufacturer’s protocol.

### 4.6. Energy Metabolism (ATP Breakdown)

The determination of ATP in liver samples homogenated in a perchloric acid solution was performed using the ATP assay kit for fluorimetry (Sigma Aldrich ATP colorimetric/fluorimetric assay kit, Madrid, Spain). The ATP concentration was determined by the phosphorylation of glycerol, which is a detectable product for the fluorimeter (excitation/emission 535 nm/587 nm) at 37 °C and proportional to the amount of ATP in the sample. Energy breakdown during cold storage was measured through the changes in ATP levels, as previously described [29].

### 4.7. Western-Blotting Analysis

Liver tissue was homogenized in HEPES buffer, and proteins were separated by sodium dodecyl sulfate polyacrylamide gel electrophoresis and transferred to polyvinylidene fluoride or nitrocellulose membranes. Membranes were immunoblotted overnight at 4 °C with antibodies against ubiquitin (U5379, Sigma-Aldrich, Madrid, Spain), Rpt1, 20S core subunits (α5/α7, β1, β5, β5i, and β7) (BML-PW 8895 and BML-PW8825, respectively, ENZO Life Sciences, Madrid, Spain), AMPK (#ab80039) and p-AMPK (#133448) (Abcam, Cambridge, UK). Antibodies against e-NOS/NOS (#610297, BD Biosciences, Madrid, Spain) and P-eNOS (#9571, Cell Signalling, Leiden, The Netherlands) were also used. Membranes were washed afterwards and incubated 1 h at room temperature with secondary antibody. Detection was performed with anti-IgG-HRP (Santa Cruz Biotechnology, Inc., Heidelberg, Germany). Signals were detected by enhanced chemiluminescence and quantified by scanning GS800 calibrated densitometer (Biorad, Madrid, Spain). Both β-actin and α-tubulin were used as loading controls.

### 4.8. Statistics

Data are expressed as mean ± standard error, and were compared statistically by variance analysis, followed by the Student–Newman–Keuls test using GraphPad Prism version 4.02 for Windows (GraphPad Prism software; Accession data: 17 May 2004). *p* < 0.05 was considered significant.

## 5. Conclusions

In conclusion, the data reported here show that IGL-1 preservation solution performs better than HTK preservation solution for fatty liver graft cold storage. The effects of this preservation may be directly related with the oncotic agent, PEG 35, contained in the IGL-1 solution, since its presence is one of the main differences between the solutions. More studies are now required in order to explore this possibility further. The protective effects of IGL-1 solution are associated, at least in part, with the activation of AMPK, stabilization of the cell ATP content and high e-NOS expression. This leads to the inhibition of the UPS activity and the ubiquitination of proteins which, in the final analysis, reduce the cold ischemia-associated damage in fatty livers.

## Figures and Tables

**Table 1:** Comparison between histidine-tryptophan-ketoglutarate (HTK) and Institute Georges Lopez (IGL-1) preservation solutions components.

Electrolyte (m·mol/L)	IGL-1	HTK	Colloids (g/L)	IGL-1	HTK
K^+^	25	9	Polyethylene glycol-35	1	
Na^+^	125	15			
Mg^2+^	5	4			
Cl^−^		50			
SO_4_^2−^	5				
Buffers (m·mol/L)			Antioxydants (m·mol/L)		
Diphosphate	25		Glutathione	3	
Histidine		180			
Histidine-HO		18	Allopurinol	1	
Tryptophan		2			
Impermeants (m·mol/L )			Precursors (m·mol/L )		
Raffinose	30		Adenosine	5	
Lactobionic acid	100		Ketoglutarate		1
Mannitol		30	pH	7.4	7.2
			Osmolarity	290	310
